# Forceful patterning: theoretical principles of mechanochemical pattern formation

**DOI:** 10.15252/embr.202357739

**Published:** 2023-11-02

**Authors:** Jan Rombouts, Jenna Elliott, Anna Erzberger

**Affiliations:** ^1^ Cell Biology and Biophysics Unit European Molecular Biology Laboratory (EMBL) Heidelberg Germany; ^2^ Developmental Biology Unit, European Molecular Biology Laboratory (EMBL) Heidelberg Germany; ^3^ Department of Physics and Astronomy Heidelberg University Heidelberg Germany

**Keywords:** geometry, mechanics, morphogenesis, pattern formation, theory

## Abstract

Biological pattern formation is essential for generating and maintaining spatial structures from the scale of a single cell to tissues and even collections of organisms. Besides biochemical interactions, there is an important role for mechanical and geometrical features in the generation of patterns. We review the theoretical principles underlying different types of mechanochemical pattern formation across spatial scales and levels of biological organization.

## Graphical abstract

Besides biochemical interactions, there is an important role for mechanical and geometrical features in the generation of biological patterns. This review discusses the theoretical principles underlying mechanochemical pattern formation across spatial scales and levels of biological organization.

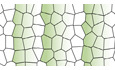

**List of symbols**
In the text, bold symbols indicate vector or tensor quantities. A subscript 0 indicates a steady‐state quantity. A tilde indicates a small perturbation from steady state. 
*x, y, z*
: space coordinates
*t*
: time coordinate
*L*
: domain length
*R*
: radius
Γ: two‐dimensional surface
*A*
: surface area
*H*
: mean curvature
λ: pattern wavelength
*q*
: wavenumber
ω
: growth rate
ℓ
: microscopic length
*c*
: concentration
*j*
: flux
*v*
: velocity
σ: stress
*f*
: external force
*h*
: height function
*r*
: displacement
ϕ
: oscillator phase
ℛ
: reaction term
*F*
: free energy
*D*
: diffusion coefficient
η
: viscosity
γ
: surface tension
τ
: timescale
α
: friction coefficient
κ
: bending rigidity
$C_{p}$
: spontaneous curvature

## Introduction

**Figure 1:**
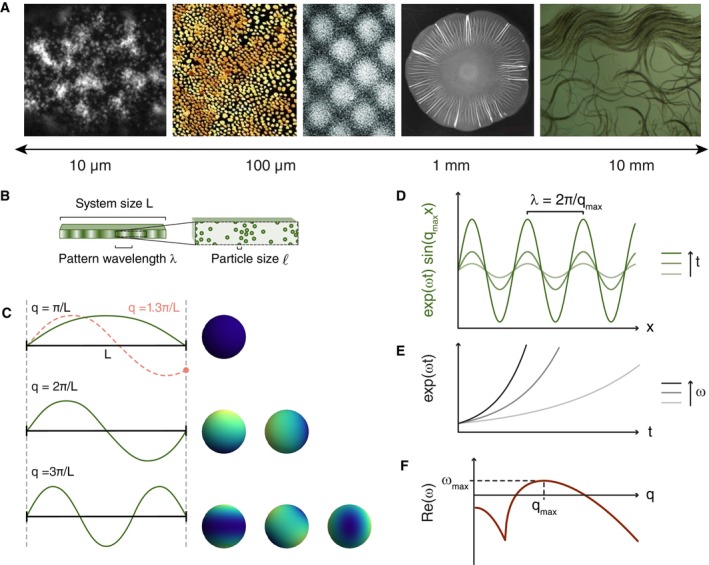
Mechanochemical pattern formation across scales (A) From left to right: Myosin patterns at the surface of *C. elegans* embryos (Reymann *et al*, 2016), ERK waves in MDCK monolayers (Lin *et al*, 2022), cell density patterns in penguin skin (Curantz *et al*, 2022), buckled biofilm (Yan *et al*, 2019), and collective configuration of nematodes (preprint: Quillen *et al*, 2021a). (B) Within a system of size L, the wavelength of a pattern λ is larger than the size of individual units ℓ. (C) A pattern in a system with size L and fixed values at the boundaries can only contain modes with wavenumbers that are integer multiples of π/L; other modes do not fulfill the boundary conditions (red dotted line). The dimension and shape of the domain matter for the spatial modes: a pattern can be expanded into a linear combination of sinusoidal modes on a one‐dimensional domain (left) and into a combination of spherical harmonic modes on a spherical domain (right). (D, E) When a field develops an instability from a uniform steady state, linear stability analysis reveals the dominant length scale $\lambda =2\pi /q_{\max}$ that appears first. This mode $q_{\max}$ has the fastest growth rate Reω, that is, it is at the maximum of the dispersion relation. (F) The dispersion relation for equation (20) reveals the temporal dynamics of different modes: regions where $\omega \left(q\right)$ is positive or negative correspond to growing or decaying modes, respectively. If there are unstable modes, that is, Reω>0 for some q, there is an instability that can lead to patterns.

The spontaneous generation of spatial structures is a hallmark of living matter that enables biological function from the molecular to the organismal scale. Indeed, patterns are some of the most conspicuous and beautiful features of the biological world. Markings on animal skins are a famous example, but there is a wide variety of systems that exhibit pattern formation. Fig 1A shows some examples in which pattern formation relies on *mechanical* processes. On the micrometer scale, patterns in the cytoskeleton at the surface of the cell emerge from active contractile stresses generated by molecular motors (Salbreux *et al*, 2012; Reymann *et al*, 2016). Tissue‐scale patterns arise from the interplay between cell signaling and mechanical cell–cell interactions (Hino *et al*, 2020; Boocock *et al*, 2021). Mechanical interactions can drive morphogenesis as exemplified by the hexagonal patterns on the skin of avian embryos that instruct feather placement (Shyer *et al*, 2017; Curantz *et al*, 2022; Palmquist *et al*, 2022). Multiple organisms form patterns on scales of millimeters to centimeters, for example, through constrained growth in bacterial biofilms (Gordon *et al*, 2017; Yan *et al*, 2019) or hydrodynamic and contact‐based interactions in collections of nematodes (Peshkov *et al*, 2022). These patterns have different functions in cellular contexts, the development and maintenance of an organism, or in the collective organization of multiple organisms. To obtain more insight into these functions, an understanding of the biochemical and mechanical aspects that control pattern formation is essential.

In the following, we introduce some of the theoretical concepts used to study pattern formation. We consider patterns as regular spatial features that consist of many units (e.g., cells or molecules), where the characteristic length scale of the pattern is much larger than the size of a single unit. A pattern can be structural, characterized by a regular shape, or it can manifest as regular variations in concentration or density. Typically, a pattern consists of *repeated* features. In that case, we identify a pattern's length scale with its wavelength, that is, the distance between repeated features. We refer to the length scale of individual units ℓ as microscopic and the pattern length scale λ as mesoscopic, relative to the macroscopic size L of the whole system (Fig 1B).

Not all details of a system's individual microscopic units are relevant for determining its mesoscopic features. When the spatial scales are well separated (i.e., when λ≫ℓ), the large number of microscopic units that make up a wavelength of the pattern permits a statistical description. An example of such a description is a concentration field that keeps track only of the average number of units per length, area, or volume at each point in space and time. A separation of spatial scales, in many cases, also implies a separation of temporal scales. Indeed, the mean collision time of water molecules within a typical ocean wave with a period of 10 s is below $10^{-10}\;s$. Within the actin cortex at the cell surface, typical timescales of macromolecular interactions are on the order of milliseconds, whereas they lead to cell‐level shape dynamics and flows on the minute‐to‐hour scale (Salbreux *et al*, 2012; Bergert *et al*, 2015; Reymann *et al*, 2016). Under such circumstances, a system can be described in the continuum limit in terms of field variables that evolve in time. The mesoscopic parameters that characterize the continuum level can be estimated from the microscopic dynamics. For example, the coarse‐grained viscoelastic properties of multicellular aggregates can be related to the microscopic parameters of cellular interactions (Oriola *et al*, 2022).

If a system is made up of a small number of constituents, or if the focus is on individual interactions, a discrete framework might be a more appropriate description. Discrete modeling frameworks can be used to study, for example, the dynamics of individual cytoskeletal filaments and associated proteins (Nedelec & Foethke, 2007) or interactions between cells (Graner & Glazier, 1992; Alt *et al*, 2017; preprint: Belousov *et al*, 2023). Additionally, discrete models are also essential to describe systems where the length scale of the pattern is similar to the length scale of the individual units (e.g., Collier *et al*, 1996; Manukyan *et al*, 2017; Fofonjka & Milinkovitch, 2021). In this paper, however, we focus on continuum approaches to pattern formation. Continuum theories have successfully predicted large‐scale flows, deformations, and patterns in many cellular and multicellular systems (Bergert *et al*, 2015; Streichan *et al*, 2018; Erzberger *et al*, 2020; Saadaoui *et al*, 2020; Palmquist *et al*, 2022).

The dynamics in space and time of mesoscale fields, such as concentrations, follow partial differential equations that are called *continuity equations*. When the fields correspond to densities of conserved quantities such as mass or momentum, the governing partial differential equations express conservation laws. For example, the concentration c of a conserved number of molecules at position x and time t evolves according to the spatial derivatives of the flux density j at that point:

(1) $$\partial_{t}c\left(x,t\right)=-\nabla \cdot j.$$

This equation states that the change in c depends on how many molecules enter and exit at a certain location. The ∇ operator can be expressed in terms of spatial derivatives, and in one dimension is equal to $\partial_{x}$. The flux density j can arise, for example, from diffusion, which for molecules usually obeys Fick's law: $j_{\text{diffusive}}=-D\nabla c$ (Fick, 1855). Plugging the diffusive flux into equation (1) leads to the familiar diffusion equation $\partial_{t}c=D\nabla^{2}c$. The same equation can be used for collections of cells, rather than molecules, that are moving about randomly (Berg, 1993).

In many biological contexts, the total number of units is not conserved. For example, cells and organisms reproduce and die, and molecules are subject to chemical reactions. Such processes lead to reaction terms $ℛ\left(c\right)$ in the equation for the corresponding density or concentration that describes how c changes locally. Such reaction–diffusion equations

(2) $$\partial_{t}c=D\nabla^{2}c+ℛ\left(c\right)$$

are classical pattern‐forming systems. The seminal work of Turing (1952) showed how biochemical feedback mechanisms combined with diffusion can lead to the emergence of regular patterns. Turing's work was conceptually extended and described in the context of activator–inhibitor systems (Gierer & Meinhardt, 1972; Meinhardt, 2012). Such systems need at least two chemical species to form patterns (see Box 1 for an example). Turing's original two‐species example is mathematically simple but has often been considered biologically unrealistic due to, for example, its sensitivity to parameter values (Green & Sharpe, 2015). Recent theoretical and computational work has shown, however, that a Turing mechanism can robustly produce patterns in more complex reaction–diffusion systems (Marcon *et al*, 2016; Haas & Goldstein, 2021).

Box 1 **Linear stability analysis of partial differential equations.**

Linear stability analysis (LSA) provides insights into the behavior of a system from its steady states and their stability. A system is in a steady state if it is not changing over time. If this state is stable, the system will return to it after being perturbed. If there exists at least one perturbation to the steady state which grows in time, leading the system away from it, the steady state is called unstable. As an example, we consider a reaction–diffusion system (see equation 2) in one dimension, the Schnakenberg system (Schnakenberg, 1979; Murray, 1982). This is a pattern‐forming system of purely chemical origin. Two chemicals A and B react and diffuse, and the evolution of their concentrations is given by

(18) $$\begin{array}{ll} \partial_{t}A & =D_{A}\partial_{\mathrm{xx}}A+k_{1}A^{2}B+k_{2}-k_{3}A,\\ \partial_{t}B & =D_{B}\partial_{\mathrm{xx}}B-k_{1}A^{2}B+k_{4}. \end{array}$$

The terms with the spatial derivatives describe diffusion with diffusion constants $D_{A}$ and $D_{B}$ and the remaining terms describe the chemical reactions. The chemical species A and B are produced at constant rates $k_{2}$ and $k_{4}$_,_ respectively. There is a conversion reaction with rate constant $k_{1}$, and A is degraded with rate constant $k_{3}$. Appropriate scaling of the variables, space, and time yields equations in terms of the dimensionless variables u and v

(19) $$\begin{array}{ll} \partial_{t}u & =\partial_{\mathrm{xx}}u+{ℛ}_{u}\left(u,v\right)=\partial_{\mathrm{xx}}u+\alpha -u+u^{2}v,\\ \partial_{t}v & =d\partial_{\mathrm{xx}}v+{ℛ}_{v}\left(u,v\right)=d\partial_{\mathrm{xx}}v+\beta -u^{2}v, \end{array}$$

with only three dimensionless parameters: $\alpha =\left(k_{1}^{1/2}k_{2}\right)/k_{3}^{3/2}$, $\beta =\left(k_{1}^{1/2}k_{4}\right)/k_{3}^{3/2}$, and the ratio of diffusion coefficients $d=D_{B}/D_{A}$.

To analyze the system, we first identify the spatially uniform steady states—for which ${ℛ}_{u}\left(u,v\right)=0$ and ${ℛ}_{v}\left(u,v\right)=0$—and then determine their stability to spatial perturbations. In real systems, such perturbations are typically due to noise. For the Schnakenberg equations (19), there is a single steady state given by $u_{0}=\alpha +\beta$ and $v_{0}=\beta /{\left(\alpha +\beta \right)}^{2}$. To analyze its stability, we derive how small perturbations from this steady state, $\tilde{u}$ and $\tilde{v}$, evolve over time. Substituting $u=u_{0}+\tilde{u}$ and $v=v_{0}+\tilde{v}$, we obtain

(20) $$\begin{array}{ll} \partial_{t}\tilde{u} & \approx \partial_{\mathrm{xx}}\tilde{u}+\partial_{u}{ℛ}_{u}\left(u_{0},v_{0}\right)\tilde{u}+\partial_{v}{ℛ}_{u}\left(u_{0},v_{0}\right)\tilde{v}\\ \partial_{t}\tilde{v} & \approx d\partial_{\mathrm{xx}}\tilde{v}+\partial_{u}{ℛ}_{v}\left(u_{0},v_{0}\right)\tilde{u}+\partial_{v}{ℛ}_{v}\left(u_{0},v_{0}\right)\tilde{v}, \end{array}$$

in which we have kept terms in linear order in the small perturbations. In this linearized version, one can already see the feedback loops that are acting in the system. For example, $\partial_{v}{ℛ}_{u}\left(u_{0},v_{0}\right)={\left(\alpha +\beta \right)}^{2}$ is positive, indicating that there is positive feedback from B to A.

This linear system can be solved for any initial condition by decomposing the initial spatial profile into a sum of spatial modes and studying the temporal evolution of these modes. The spatial modes that are appropriate depend on the geometry of the system. In this one‐dimensional example, suitable spatial modes are the functions $\cos\left(\mathrm{qx}\right)$ and $\sin\left(\mathrm{qx}\right)$ for different wavenumbers q. As known from Fourier analysis, a profile can be decomposed into combinations of these functions. These spatial modes are appropriate because their shape (but not their amplitude) is unchanged when applying the diffusion operator $\partial_{\mathrm{xx}}$. This, together with the linearity of the equation, allows to study the evolution of equations (20) by solely considering what happens to the spatial modes. The temporal evolution of an initially sinusoidal profile is given by $e^{\mathrm{\omega t}}\sin\left(\mathrm{qx}\right)$ and $e^{\mathrm{\omega t}}\cos\left(\mathrm{qx}\right)$. Each of the modes’ amplitude thus evolves as $e^{\mathrm{\omega t}}$ (Fig 1D and E). The value of ω depends on the wavenumber q. The relation $\omega \left(q\right)$ is called the *dispersion relation* and can be computed from equations (20). This relation describes the growth rates of the different spatial modes (Fig 1F). Generally, $\omega \left(q\right)$ is a complex number. Its real part describes the growth of the mode: perturbations with Reω<0 decay in time, leaving the uniform solution intact. However, if for a given q, one of the growth rates has a positive real part; the amplitude of the perturbation $e^{\mathrm{\omega t}}$ grows in time. This implies that the uniform steady state is unstable to perturbations with wavenumber q. The formation of patterns is associated with the existence of non‐zero wavenumbers that have Reω>0. The fastest‐growing mode—at the maximum of the dispersion relation—dominates, and thus sets the initial spatial wavelength of the pattern. If $\omega \left(q\right)$ has an imaginary part, the solution is oscillatory over time. A mode that is growing in amplitude and is oscillatory leads to spatiotemporal patterns such as waves, whereas a purely real ω is associated with stationary patterns. The dispersion relation of the Schnakenberg system reveals the conditions on α,β, and d that lead to pattern formation, that is, for which values of these parameters unstable modes with q>0 exist.

The geometry and size of the system impose further conditions on the solutions, mainly by constraining the form of the spatial modes and available wavenumbers. The sinusoidal form of the spatial modes is imposed by the fact that we study a one‐dimensional system. The particular wavenumbers that are possible are dictated by the domain size and boundaries: if the chemical reactions of the Schnakenberg system happen on a closed interval $\left[0,L\right]$ with either fixed concentrations or no‐flux conditions at the boundaries, the only possible wavenumbers are of the form q=nπ/L, with an integer n. Patterns appear only when one of the permitted non‐zero modes has a positive growth rate. The size of the domain thus restricts which wavenumbers are possible. Analogously, other domain shapes and sizes dictate the spatial modes that can become unstable. This is illustrated in Fig 1C.

Since we assumed small perturbations to the steady state in going from equations (19) to (20), the linear stability analysis only reveals the dynamics of systems that are close to a steady state. Once the perturbation becomes too large, non‐linear effects come into play and analytical results are hard to obtain. Moreover, non‐linear terms in the equations dictate at which amplitude the growing perturbations saturate, and also determine which types of patterns appear; for example, whether spots or stripes appear in two‐dimensional systems. Finally, we note that not all patterns exist close to a homogeneous steady state—patterns may also exist “far from threshold” and depend crucially on the non‐linearities in the system. We refer to the book by Cross & Greenside (2009) for more details about these aspects. Even with these caveats, in many cases, the linear stability analysis already provides good insight into the system's pattern‐forming properties and leads to an understanding of which biological parameters determine the existence and characteristic scale of patterns, as we illustrate throughout this review.

In most biological systems, patterns are not due to biochemical or genetic interactions alone. Mechanical forces play an important role. The propagation of mechanical stresses occurs on different temporal and spatial scales than molecular diffusion. For example, a small molecule requires seconds to diffuse over a 10‐100 µm distance, whereas mechanical stress propagates over a similar distance within microseconds in the cytoskeleton (Wang *et al*, 2009). Mechanical or mechanochemical pattern formation thus broadens the range of scales on which patterns can be formed (Howard *et al*, 2011; Collinet & Lecuit, 2021). In fact, since all organisms and cells are subject to physical laws, the effects of these laws are likely exploited for the generation of patterns with biological functions.

An aspect that is inherently linked to mechanics is the size and geometry of the domain on which these patterns form, such as the shape of cell membranes or tissue layers. Spatial derivative operators, such as ∇, take different forms depending on the dimension of the system, its geometric properties, and the chosen coordinate system. This, in turn, influences the types of spatial modes that are used in a linear stability analysis (see Box 1) and the patterns that can appear. Moreover, the shape of the domain can influence the dynamics of the pattern: Nishide & Ishihara (2022) recently showed that Turing‐type patterns that are static on a flat domain can propagate on curved shapes.

Furthermore, mechanical forces affect the flux term in equation (1). For example, if there are fluid flows in the system that carry along molecules or cells, there is a contribution of an advective flux $j_{\text{advective}}=cv$, where v is the velocity field. The evolution of v is governed by a second equation that states the conservation of momentum. This equation can be derived in the same way as equation (1), but luckily, it can be simplified for many biological contexts by neglecting the time‐derivative term on the left‐hand side because, on the scale of cells and small organisms, forces due to friction, or viscosity, dominate over inertial forces. Such *overdamped* motion appears very different from our everyday experiences (Purcell, 1977; Lauga & Powers, 2009). In this limit, the conservation law for momentum can be written as

(3) $$0=\nabla \cdot \sigma_{\mathrm{tot}}+f,$$

in which f denotes any external forces, such as those arising from friction with the environment. Equation (3) is a continuity equation like equation (1), where the time derivative of the velocity on the left‐hand side is zero. In the same way in which we keep track of how particles enter and leave a location in equation (1), we must account for the transport of momentum through a flux term. The counterpart of j in equation (1) is written as the negative momentum flux density by convention and is called the total stress tensor $\sigma_{\mathrm{tot}}$. It describes all forces per area that act on a small piece of the material for each of the possible force directions. Same as for the molecular flux density, the components of the stress tensor depend on the properties of the system. In an incompressible Newtonian fluid, for example, a viscous stress arises between adjacent fluid layers that is proportional to the velocity difference between the layers. Additionally, in most biological systems, there are active contributions to the stress, $\sigma_{\text{active}}$ (Kruse *et al*, 2005; Jülicher *et al*, 2018), generated by processes such as the ATP‐fuelled motion of molecular motors within the cytoskeleton (Bois *et al*, 2011; Dasanayake *et al*, 2011; Peleg *et al*, 2011), or the self‐propelled movement of migrating cells or swimming microorganisms (Yeomans, 2017; Alert & Trepat, 2020). Such active processes are characteristic of biological systems and can give rise to behavior not seen in passive materials. This aspect of living systems motivated many successful developments within the research field of active matter physics, with relevance beyond biology (Ramaswamy, 2010; Marchetti *et al*, 2013; Needleman & Dogic, 2017; Bowick *et al*, 2022).

An equation for force balance can also be derived starting from energy‐minimization considerations, where a force is induced by a system moving toward configurations of lower energy (see, e.g., section “Pattern formation by membrane curvature”).

Once the governing equations of a system have been determined, we can analyze whether they admit pattern formation. One way of doing this is to simulate the time evolution of the equation on a computer and see whether patterns appear over time. Additionally, an analytical method called linear stability analysis is useful to obtain conditions on pattern formation and estimates of typical pattern length scales (see Box 1, or the book by Murray (2003) for a more detailed explanation).

Pattern formation typically arises due to different kinds of feedback mechanisms between the variables of the system. Positive feedback implies a self‐reinforcing coupling between chemical or mechanical variables which leads to the amplification of small perturbations over time, whereas negative feedback inhibits the growth of perturbations. Typically, both positive and negative feedbacks are present in pattern‐forming systems: positive feedback amplifies perturbations that lead to patterns, but negative feedback prevents these from increasing unboundedly. Additionally, negative feedbacks are often associated with oscillatory dynamics, which in spatially extended systems can lead to waves (see Beta & Kruse (2017) and section “Waves through mechanosensing”).

In the following, we review some of the main patterning phenomena involving mechanics and shape, and focus on the underlying theoretical principles. We selected examples in which the outlined continuum framework and linear stability analysis can be applied, and mainly consider patterns that arise from the instability of a uniform steady state. We begin by discussing the role of geometry in the development of protein patterns at the surface of cells, covering aspects such as bulk‐surface coupling and membrane–curvature interactions. We then discuss patterns generated by contractile instabilities, where active contractile elements produce self‐convecting flows. The universal nature of this pattern‐forming mechanism is highlighted by its relevance both at the subcellular and at the multicellular level. Next, we briefly discuss purely mechanical interactions such as buckling, and how these can lead to periodic patterning. Finally, we discuss waves, which are examples of patterns that show both temporal and spatial variation. We describe two different types of waves: one mechano‐chemical and one based on the synchronization of oscillators due to hydrodynamic effects. The selected examples are ordered roughly along increasing spatial scales and levels of biological organization from subcellular to organismal.

## Geometry‐dependent reaction–diffusion patterns

Pattern formation at the cell surface is coupled to shape and curvature and is influenced by the biophysical properties of the cell membrane and the cytoskeleton. In particular, reaction–diffusion dynamics in cells often involve an exchange of components between structures at the cell surface, that is, the plasma membrane or the actomyosin cortex, and the cytosol. These fluxes give rise to biochemical couplings between nearly two‐dimensional structures that can be approximated as surfaces and a three‐dimensional bulk. Generally, the three‐dimensional geometry of a cell has an important influence on biochemical reactions, both in the cytoplasm and on cell membranes. The dependence of biochemical patterns on cellular geometry enables shape sensing: the process by which shape information is incorporated into, and used to instruct, cellular processes.

**Figure 2:**
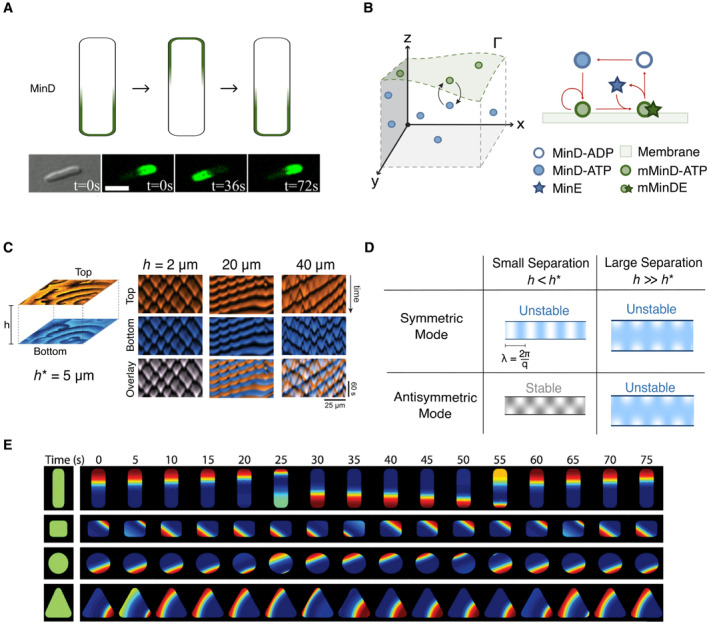
Min patterns can sense cell shape (A) Sketch of MinD oscillation (top) and images of MinD in *E. coli*, showing oscillations in a live cell (bottom), adapted from Bonny *et al* (2013). Scale bar: 3μm. (B) Schematic representations show the compartment geometry (left) with components of the Min reaction–diffusion system in the cytosol (blue) and bound to the surface Γ (green), and a diagram of a minimal Min reaction model (right), adapted from Meindlhumer *et al* (2023). (C) Setup of the double‐membrane configuration (left). A top membrane (orange) is separated by a cytosolic volume with height h from a lower membrane (blue). The observed patterns change as h is varied. Kymographs from simulations show a decrease in the coupling between patterns at the two surfaces for increasing separation heights (right), where $h^{*}=5\mu m$ is the critical height above which non‐standing wave patterns emerge. Adapted from Brauns *et al* (2021). (D) Sketches visualizing the unstable (blue) and stable (gray) perturbation modes of a single wavenumber q at large and small membrane separations. For small separations, the antisymmetric mode is stable and therefore does not contribute to pattern emergence. (E) Simulation results showing Min oscillations along symmetry axes of confining shapes (from Wettmann *et al*, 2018).

Such a shape‐sensing mechanism has been proposed as the function of certain protein patterns at the cell surface. For example, Min proteins undergo patterning at the surface of *E. coli* bacteria (de Boer *et al*, 1989; Wettmann & Kruse, 2018; Fig 2A). Here, a standing wave oscillation in the concentrations of Min proteins provides a means of localizing the center of the cell (de Boer *et al*, 1989; Lutkenhaus, 2007). This standing wave templates the formation of a contractile protein ring to ensure symmetric cell division.

The spatial distribution of Min proteins evolves according to coupled reaction–diffusion equations that consist of both cytosolic and membrane‐bound components. Early observations of this system in division‐inhibited bacteria showed that the concentration of a membrane‐bound Min protein formed standing waves in elongated cells (Hu & Lutkenhaus, 1999; Raskin & de Boer, 1999), and such waves were later also observed *in vitro* in closed geometries (Zieske & Schwille, 2013). In comparison, on flat membranes, the Min system produces traveling and spiral waves and many other pattern structures (Loose *et al*, 2008; Ivanov & Mizuuchi, 2010; Denk *et al*, 2018).

A brief form of the reaction–diffusion system can be written in terms of a vector of cytosolic components c and a vector of membrane‐bound components m (Huang *et al*, 2003; Halatek & Frey, 2012). The cytosolic molecules diffuse in the three‐dimensional bulk with coefficient $D_{c}$, and the membrane‐bound molecules diffuse on the two‐dimensional boundary surface Γ with coefficient $D_{m}$. The equations read

(4) $$\partial_{t}c=D_{c}\nabla_{3D}^{2}c+{ℛ}_{c}\left(c\right),$$

(5) $$\partial_{t}m=D_{m}\nabla_{2D}^{2}m+{ℛ}_{m}\left({\left.c\right|}_{\Gamma },m\right).$$

The reaction terms ${ℛ}_{c}$ and ${ℛ}_{m}$ describe the chemical reactions in the cytosol and between bulk and surface components, where ${\left.c\right|}_{\Gamma }$ indicates the cytosolic concentrations at the membrane–cytosol boundary. A minimal reaction schematic for these reactions is depicted in Fig 2B.

The dynamics in the bulk are coupled to those on the surface through a boundary condition accounting for particle conservation: the flux of particles leaving and entering the cytosol is balanced by the rates of membrane binding and unbinding. Modeling details, including cooperative binding (Loose *et al*, 2008), further biochemical reactions (Meinhardt & Boer, 2001; Loose *et al*, 2011; Park *et al*, 2011; Ayed *et al*, 2017; preprint: Carlquist & Cytrynbaum, 2021), protein polymerization (Drew *et al*, 2005; Cytrynbaum & Marshall, 2007), interactions with particular lipids localized at the cell poles (Renner & Weibel, 2012), and advective cytoplasmic flows (Vecchiarelli *et al*, 2014; Meindlhumer *et al*, 2023), have been extensively studied and are proposed to play important roles in regulating Min patterns (Vecchiarelli *et al*, 2016; Wettmann & Kruse, 2018; Ramm *et al*, 2019; Takada *et al*, 2022).

The Min reaction system includes an ATPase, MinD, that enhances the recruitment of itself to the membrane and a second protein, MinE, which drives MinD off the membrane again (Wettmann & Kruse, 2018; Ramm *et al*, 2019). Under specific *in vitro* conditions, such systems have been shown to exhibit stationary patterns (Glock *et al*, 2019), but most research focuses on their emergent oscillatory dynamics. These have been proposed to arise from a difference in the time MinD and MinE spend in the cytosol before re‐binding to the membrane, introduced by the time needed for nucleotide replenishment of the ATPase MinD (Huang *et al*, 2003; Glock *et al*, 2019). Whereas *in vitro*, this system admits different types of oscillating patterns, including both traveling waves and standing waves, *in vivo* cells robustly produce standing waves (Fig 2A; Hu & Lutkenhaus, 1999; Raskin & de Boer, 1999; Bonny *et al*, 2013). Different groups have shown that the geometry of the cell may play a crucial role in the robust selection of standing wave patterns (Schweizer *et al*, 2012; Zieske & Schwille, 2013, 2014; Wu *et al*, 2015). Although the exact mechanism behind the emergence of patterns in different geometries is still under debate (Park *et al*, 2011; Halatek & Frey, 2012; Halatek *et al*, 2018; Wettmann & Kruse, 2018; Ramm *et al*, 2019; preprint: Carlquist & Cytrynbaum, 2021; Takada *et al*, 2022), protein depletion has been suggested to play an important role in the formation of standing waves (Vecchiarelli *et al*, 2016), and Brauns *et al* (2021) propose that this arises due to the narrow geometry of the cells. The boundary condition for the cytosolic concentrations enforces protein conservation. In a small cytosolic volume, such as in the narrow *in vivo* geometry, the flux of particles from the cytosol onto the membrane leads to cytosolic protein depletion.

To understand how changes in system geometry can lead to transitions between patterning regimes, Brauns *et al* (2021) considered a particular configuration in which two flat, extended membranes are separated by a variable volume of cytosol (Fig 2C). As discussed in Box 1, the size and shape of the patterning domain determines which perturbation modes are available to a system. Here, the geometric configuration restricts the perturbations to be periodic parallel to the membrane, and either symmetric or asymmetric in the perpendicular direction (Fig 2D). Crucially, linear stability analysis shows that it is the separation distance between the two membranes that determines which of these perpendicular modes grow over time, and what type of patterns the system supports. This introduces a dependence on geometry to the patterning process. Below a critical value of membrane separation, only the symmetric mode perturbations undergo growth. This results in the same cytosolic concentration dynamics at the two membranes and leads to standing wave patterns.

Above the membrane separation threshold, the antisymmetric modes also grow in time and therefore the protein concentrations at the two membranes are no longer constrained to be equal. In this regime, many more perturbation modes exhibit growth and the interactions with a bulk supply of proteins in the cytosol can lead to the emergence of many different types of patterns.

The geometry of the boundaries thus controls the type of patterns that appear in the Min system. Brauns *et al* (2021) confirmed these predictions qualitatively through simulations (Fig 2C) and in experiments, using a microfluidic system with variable chamber height. These results suggest that *in vivo*, the narrow radius of the cylindrical bacteria provides the geometrical constraint on Min patterns that allows the formation of standing waves.

The Min system is a classic and accessible pattern‐forming system. This is one of the reasons it is often used as a testing ground for theories on (biological) pattern formation, also beyond linear stability theory (Halatek & Frey, 2018; Brauns *et al*, 2020; Würthner *et al*, 2022). This system is also an example of a “mass‐conserving reaction diffusion system,” where spatial redistribution, such as protein depletion, drives the emergence of protein patterns. For a more detailed discussion of these systems, and their comparisons with non‐mass conserving systems, see Halatek *et al* (2018).

In elongated, rod‐like cells such as *E. coli*, the standing waves produced by the Min system provide a mechanism to read out the mid‐line and determine the cell division plane. A natural question is then which Min patterns form in non‐rod‐like cells. Wu *et al* (2015) put *E. coli* cells in microchambers with different shapes, and observed that Min patterns oscillated along one of the symmetry axes. Subsequent simulations by Wettmann *et al* (2018) have also matched these results (Fig 2E). Walsh *et al* (2019) studied archaeal cells, where the division plane is also determined by a Min system. They focused on nearly triangular cells in particular and observed that the division plane corresponds to what is predicted by an analysis of the spatial modes determined by the cell shape. These examples also show how shape is a fundamental property that can guide the biochemical machinery of the cell.

Min patterns thus respond to global properties of cell shape. By contrast, patterns at the surface of starfish oocytes have been proposed to act as “local” shape sensors (Wigbers *et al*, 2021). In these cells, the angle of the membrane relative to the direction to the nucleus, and its distance from the nucleus, are sensed through a protein concentration gradient. Similarly to the embryos of several other species (Sawai, 1982; Yoneda *et al*, 1982; Quaas & Wylie, 2002), starfish oocytes feature a contraction wave along their membrane before cell division, which starts at the vegetal pole (far from the nucleus) and travels to the animal pole (close to the nucleus; Hamaguchi & Hiramoto, 1978; Klughammer *et al*, 2018). This traveling wave ends at the animal pole regardless of the oocyte shape, implying a modulation of the wave speed according to local cell shape (Bischof *et al*, 2017). Wigbers *et al* (2021) proposed a speed modulation mechanism for this traveling wave in the form of a temporally decaying protein concentration profile in the cytosol. The concentration has a maximum at the nucleus and decreases linearly with distance away from it. If the nucleus is not perfectly centered in the cell, the protein concentration will be different at different points on the cell surface. This links the cell's biochemistry to its geometry. The protein concentration's temporal decay at the membrane then guides the progression of the contractile wave and forces the wave speed to adapt to local membrane geometry. Together with the fact that the cell is closed, this ensures that the wave always ends at the animal pole. This system thus provides a means of local shape sensing.

Min patterns and surface contraction waves in oocytes both show how biochemical reactions may couple to cell geometry through interactions between the three‐dimensional cytosol and the two‐dimensional membrane. Overall, these examples demonstrate how biochemical reaction–diffusion systems are influenced in important ways by the geometric features of the cell.

## Pattern formation by membrane curvature

In the previous section, we discussed how the patterning dynamics of biochemical reaction–diffusion systems are modulated by the geometry of the system. Many cellular patterning processes, however, rely on two‐way interactions between the biochemical reactions and the biophysical properties of the structures they take place on (Schamberger *et al*, 2023). In this section, we discuss how membrane biophysics “by itself” can drive protein patterning. In particular, the localization of proteins to and/or transport of proteins on the plasma membrane can depend on the curvature of the membrane (Heinrich *et al*, 2010; Sorre *et al*, 2012; Kwiecinski *et al*, 2017). Additionally, membrane–protein interactions modulate the local biophysical properties of the membrane, giving rise to feedback effects that can drive pattern formation (Cooke & Deserno, 2006; Reynwar *et al*, 2007; Yue *et al*, 2010; Ramakrishnan *et al*, 2014; Sodt & Pastor, 2014; Hossein & Deserno, 2020). For example, curvature‐mediated pattern formation plays a role in the formation of cellular protrusions, such as those involved in cell migration or myelination‐like coiling. There, proteins such as curved BAR proteins couple to membrane shape and interact with the actin cytoskeleton, producing dynamic patterns and protrusions on the cell surface (Simunovic *et al*, 2015; Carman & Dominguez, 2018; Gov, 2018; Wu *et al*, 2018; Begemann *et al*, 2019; Sadhu *et al*, 2021, 2023; Ravid *et al*, 2023).

On length scales much larger than its thickness, a membrane can be approximated as a two‐dimensional sheet with a resistance to bending (Ramakrishnan *et al*, 2014; Deserno, 2015). This resistance is a mesoscopic biophysical consequence of the microscopic phospholipid structure of the layer (Bloom *et al*, 1991; Alberts *et al*, 2002): bending the membrane causes the lipid heads on one side of the bilayer to be pushed together, and to be pulled apart on the other, which results in an energetic cost for high curvatures (Würger, 2000; Kurtisovski *et al*, 2007).

**Figure 3:**
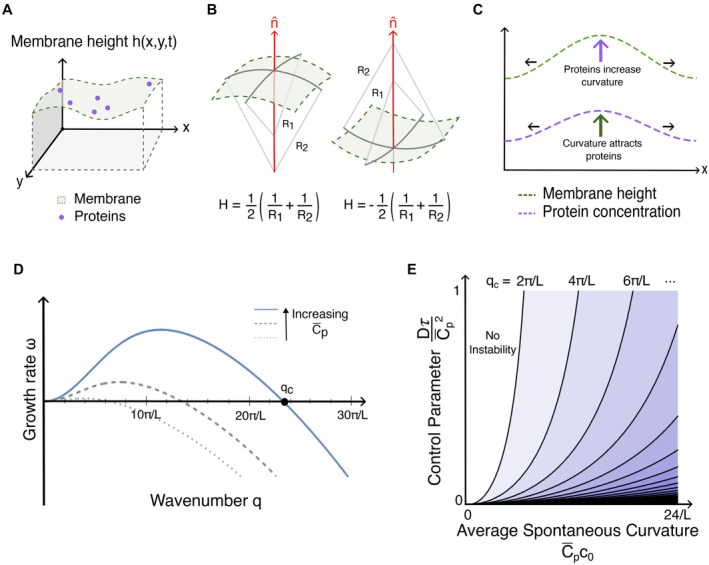
Curvature‐mediated pattern formation (A) Schematic of a membrane where the shape is described by a height function $h\left(x,y,t\right)$. Curvature‐inducing proteins are depicted in purple. The proteins can impose a non‐zero spontaneous curvature on the membrane. (B) The mean curvature H is the mean of the two principal curvatures, $1/R_{1}$ and $1/R_{2}$, of the membrane at a given point. The sign convention is chosen such that membranes curving away from the normal of the membrane (red arrow) have a positive curvature, where the normal vector is defined as positive when pointing out of the cytosolic volume. (C) Schematic of patterning mechanism for curvature‐mediated pattern formation. Membrane bending resistance and protein diffusion are counteracted by a positive feedback loop where high protein concentrations increase the local membrane curvature, which in turn attracts more proteins to the region. (D) Sketch of the dispersion relation derived from equations (9a) and (9b), relating the growth rate ω to wavenumber q, for a system of size L. The uniform state is unstable against perturbations with wavenumbers that have positive ω. The critical wavenumber $q_{c}$ corresponds to the highest wavenumber for which the uniform state is unstable (black dot). The gray dashed and dotted lines indicate how the dispersion relation changes upon changes in ${\bar{C}}_{p}$. (E) Stability diagram for curvature‐induced patterning on a domain of size L with periodic boundary conditions. The dimensionless control parameter $\frac{\mathrm{D\tau}}{{\bar{C}}_{P}^{2}}$, which indicates the relative strength of diffusive transport compared with curvature‐driven transport, is plotted against the average spontaneous curvature ${\bar{C}}_{p}c_{0}$. Contours for the critical wavenumber, $q_{c}$, are plotted in black. To the left of the line corresponding to $q_{c}=\frac{2\pi }{L}$, the uniform state is stable. To the right of the line corresponding to $q_{c}=\frac{2\pi }{L}$, the $q=\frac{2\pi }{L}$ mode becomes unstable, to the right of the $q_{c}=\frac{4\pi }{L}$ line both the $q=\frac{2\pi }{L}$ and $q=\frac{4\pi }{L}$ modes are unstable, etc.

The curvature of a two‐dimensional sheet at a given point can be described completely by its two principal radii of curvature, $R_{1}$ and $R_{2}$, which in turn define the mean curvature, H (Fig 3B). The membrane bending energy written to quadratic order in the curvature is called the Helfrich Hamiltonian (Canham, 1970; Helfrich, 1973; Deserno *et al*, 2014; Tu & Ou‐Yang, 2014; Guckenberger & Gekle, 2017; Alimohamadi & Rangamani, 2018). For membranes with no holes and negligible edge effects, it takes the form

(6) $$F_{\text{Bending}}=\int \mathrm{dA}\;\frac{1}{2}\kappa {\left(2H-C_{p}\right)}^{2},$$

which integrates the squared deviations of the mean membrane curvature H (Fig 3B) from a preferred value $C_{p}$ over the membrane area A. The bending rigidity κ characterizes the resistance of the membrane to bending (Dimova, 2014; Doktorova *et al*, 2017; Doskocz *et al*, 2018), and is related to the membrane persistence length (De Gennes & Taupin, 1982; Peliti & Leibler, 1985; Gutjahr *et al*, 2006). Here, for simplicity, we consider a system where surface tension and other energetic contributions have been neglected (see Agudo‐Canalejo & Golestanian, 2017; Tozzi *et al*, 2019, for examples where further energetic contributions have been considered).

The energetically most favorable membrane shape minimizes an energy functional F such as equation (6). For shapes described by a height function $h\left(x,y,t\right)$ (Fig 3A), this equilibrium shape is the solution to the equation 0=δF/δh, and it would be observed in a system at rest. The variational derivative δF/δh describes how a functional such as the bending energy changes for different shape functions h. Membrane shape *dynamics* arise when the system does not reside in this preferred configuration. Then, a restoring force that is proportional to δF/δh close to equilibrium drives the membrane toward the preferred configuration. This term may compete with other forces acting on the membrane. With the inclusion of frictional forces, a force balance equation (equation 3) governing the membrane shape dynamics can be written as

(7) $$\alpha \partial_{t}h=-\frac{\mathrm{\delta F}}{\mathrm{\delta h}}.$$

Here, the left‐hand side describes the frictional force on the membrane due to movement, with friction coefficient α, and the right‐hand side corresponds to the forces arising from the free energy gradient. We continue to consider a simple theory for demonstration purposes, where we have neglected the hydrodynamic effects on the system. In general, we would have to take into account flows in the membrane and the fluid surrounding it, requiring a more involved analysis (see, e.g., Shlomovitz & Gov (2009) and Frey & Idema (2021)).

For lipid bilayers with up‐down symmetry, that is, where both leaflets have the same lipid composition, the spontaneous curvature $C_{p}$ is zero. Then, the equilibrium shape that minimizes the bending energy given by equation (6)—assuming suitable boundary conditions—corresponds to a flat membrane. However, differences in bilayer composition and—importantly—interactions with proteins can lead to a non‐zero spontaneous curvature (Leibler, 1986; Lipowsky *et al*, 1998; Zimmerberg & Kozlov, 2006; Yue *et al*, 2010; Sorre *et al*, 2012; Sodt & Pastor, 2014).

The influence of protein–membrane interactions on membrane curvature has been studied across scales through numerical simulations (Reynwar *et al*, 2007; Gov, 2018), experiments (Mim & Unger, 2012), and through the development of effective field theories (Weikl *et al*, 1998; Müller *et al*, 2005; Haselwandter & Wingreen, 2014; Haussman & Deserno, 2014; Yolcu *et al*, 2014; Barakat & Squires, 2022). These works have suggested that proteins are able to impose a local change of curvature onto a membrane. At the scale of single proteins, this can lead to attractive and repulsive interactions between proteins due to the curvature of the membrane between them, with a characteristic length scale defined by the bending rigidity—typically on the order of 10 nm in cells (Johannes *et al*, 2018). At larger scales, however, the local change of curvature imposed by the proteins can be modeled as a spontaneous membrane curvature $C_{p}$ dependent on the concentration of membrane‐interacting proteins: $C_{p}=C_{p}\left(c\right)$ (Leibler, 1986; Bassereau *et al*, 2014; Argudo *et al*, 2016; Gov, 2018; Dymond, 2021). The characteristic length scale of the resulting membrane shape is significantly larger than the length scale of the individual protein–protein interactions. It depends on the bending rigidity of the membrane, the induced curvature per protein, and the diffusive properties of the proteins.

The dependence of the spontaneous curvature on the local protein concentration leads to a flux term $j_{\text{curv}}$ in the continuity equation for the protein concentration (equation 1) (Ramaswamy *et al*, 2000; Agudo‐Canalejo & Golestanian, 2017)

(8) $$\partial_{t}c=D\nabla^{2}c+ℛ\left(c\right)-\nabla \cdot j_{\text{curv}}.$$

This term arises from the coupling of protein concentrations to local membrane shape and depends on the variational derivative of the free energy with respect to the protein distribution: $j_{\text{curv}}\propto -\nabla \left(\mathrm{\delta F}/\mathrm{\delta c}\right)$ (Mahapatra *et al*, 2021).

The coupling between curvature and protein distribution can lead to the emergence of patterns. The conditions for pattern formation in the coupled system given by equations (7) and (8), with the free energy given by equation (6), can be obtained by linear stability analysis (see Box 1). To see how the concentration‐dependent spontaneous curvature by itself can drive patterning, we consider a system without chemical reactions, that is, ℛ=0 (Agudo‐Canalejo & Golestanian, 2017), and with a linear dependence of the spontaneous curvature on protein concentration $C_{p}\left(c\left(x\right)\right)={\bar{C}}_{p}c\left(x\right)$, where ${\bar{C}}_{p}$ is positive. An increase in protein concentration thus induces an increase in spontaneous curvature, which affects membrane shape, which in turn affects the protein distribution.

The equations for the steady state are solved by a uniform protein concentration $c_{0}$ and a corresponding flat equilibrium shape $h_{0}$. Linearizing around this steady state in the small gradient limit—corresponding to weak bending of the membrane—results in the equations for small perturbations $\tilde{c}\left(x\right)$ and $\tilde{h}\left(x\right)$:

(9a) $$\alpha \partial_{t}\tilde{h}=-\left(\kappa \nabla^{4}+\frac{\kappa {{\bar{C}}_{p}}^{2}c_{0}^{2}}{2}\nabla^{2}\right)\tilde{h}-\left(\kappa {\bar{C}}_{p}\nabla^{2}\right)\tilde{c},$$

(9b) $$\tau \partial_{t}\tilde{c}=\left(\mathrm{\tau D}+{{\bar{C}}_{p}}^{2}\right)\nabla^{2}\tilde{c}+\left({\bar{C}}_{p}\nabla^{4}\right)\tilde{h}.$$

Here, τ is a timescale dependent on friction, and inversely proportional to the bending rigidity. The last term in each of these equations describes the protein–membrane feedback loop, whose strength depends on the spontaneous curvature induced per protein in the membrane, ${\bar{C}}_{p}$. From these equations, we can get an understanding of the curvature‐patterning mechanism (see Fig 3C). The first term on the right side of equation (9a) accounts for the membrane's resistance to bending. The cross‐term accounts for an increase in membrane curvature caused by the curvature‐inducing proteins. The first term on the right of equation (9b) is a diffusion term that spreads the distribution of proteins. This is countered by the cross‐term which attracts proteins to regions of high positive curvature. Together, these two coupled equations describe a positive feedback loop that drives pattern formation.

From the linearized equations, we obtain the dispersion relation (Fig 3D). This shows that modes with wavenumber lower than the critical wavenumber $q_{c}={\bar{C}}_{p}c_{0}\sqrt{\frac{1}{2}+\frac{{{\bar{C}}_{p}}^{2}}{2\mathrm{\tau D}}}$ are unstable. For a membrane of infinite size, the wavenumbers may take any value. However, for systems of finite size L with periodic boundaries, the wavenumbers are constrained to integer multiples of $\frac{2\pi }{L}$. This implies that for finite‐sized systems there is no patterning if $q_{c}<\frac{2\pi }{L}$ (Fig 3E). The size of the domain thus determines whether a pattern can form. The critical wavenumber increases with ${\bar{C}}_{p}$: stronger coupling leads to more modes becoming unstable (Fig 3D). The wavenumber with the maximum growth rate, which dominates at the onset of the instability, also increases monotonically with ${\bar{C}}_{p}$. A similar analysis can be done for a closed near‐spherical geometry such as the surface of a cell (Agudo‐Canalejo & Golestanian, 2017). Although the model discussed here is too simplistic to be directly applicable in biological systems, similar mechanisms have been proposed for the distribution of mechanosensitive Piezo1 ion channels (Yang *et al*, 2022), and curvature‐sensing proteins have been shown to play a role *in vivo* in neuronal cell migration (Guerrier *et al*, 2009) and immunological cell function (Koduru *et al*, 2010).

Models similar to the one described above have also been studied in different geometries, for example, to explain the regular patterning of FtsZ proteins in bacteria (Shlomovitz & Gov, 2009) and, when coupled to active forces, to explain the formation of membrane waves (Shlomovitz & Gov, 2007; Peleg *et al*, 2011; Gov, 2018).

In summary, whereas purely chemical systems need multiple components for pattern formation (see section “Introduction”), a single species of diffusing molecules can lead to pattern formation when coupled to membrane mechanics. In the example we have discussed, the local curvature induced by a protein reduces the membrane bending energy associated with other proteins close by. Thus, an increase in local curvature will lead to an increase in the flux of curvature‐inducing proteins into the region, providing a positive feedback loop that clusters curvature‐inducing proteins, accompanied by a further increase in the local curvature. Further constraints on the membrane, such as incompressible enclosed volumes, may play a role in mode selection and the formation of stable protein patterns (Ramaswamy *et al*, 2000; Tozzi *et al*, 2019). Membrane tension, in particular, acts in opposition to increases in membrane curvature and provides a biophysical means to tune the wavelength of emergent patterns (Agudo‐Canalejo & Golestanian, 2017). Many of the results discussed in this section are valid in the limit of small membrane deformations, and it remains an interesting problem for future research to model curvature‐mediated patterning in the large deformation limit (see Box 2).

Box 2 **In need of answers.**

1. How did biological pattern‐forming systems evolve? How was their evolution facilitated or constrained by physical factors such as mechanics and geometry?

2. What biological functions are driven by mechanochemical patterning?

3. How to study—analytically and numerically—pattern formation on domains that undergo *large* deformations and/or topological changes?

4. Can we harness principles of mechanochemical pattern formation to build or control artificial systems?

In the biological context, curvature‐coupled proteins do not operate in isolation. Commonly, curvature‐sensing proteins comprise part of larger biochemical networks. In such systems, curvature coupling may provide additional feedback loops and give rise to rich patterning dynamics. For example, the inclusion of membrane–curvature coupled interactions has been suggested to enhance the robustness of biochemical pattern formation against stochastic fluctuations (Liu *et al*, 2009). Therefore, curvature‐driven patterns not only allow for cells to break symmetry and develop structural patterns but may also protect these patterns from environmental and system variability (Cail & Drubin, 2023). Moreover, in most cellular contexts, the plasma membrane is coupled to adjacent cytoskeletal components, which typically dominate the shape dynamics on length scales of 1‐10 µm. In animal cells, a meshwork of actin filaments crosslinked by various proteins, including myosin motors, forms beneath the plasma membrane and controls cellular shape changes associated with, for example, migration and division (Kelkar *et al*, 2020). Many of these essential cellular processes can be explained by considering pattern formation within the cell cortex, which we discuss in the following section.

## Contractile fluid patterning

Many living systems can generate active stresses at mesoscopic scales from microscopic processes that consume ATP or other fuel molecules. The myosin motors in the actin cytoskeleton, for example, transduce metabolic energy into work by stepping along or moving the filaments they are bound to. The activity of such motors gives rise to large‐scale and typically contractile active stresses within the material.

**Figure 4:**
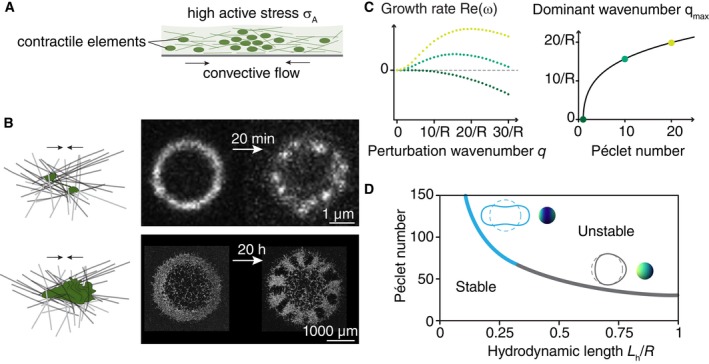
Contractile fluid patterning (A) Particles generating an active contractile stress $\sigma_{A}$ within a viscous environment drive spontaneous patterning by inducing convective flows into regions of large concentration. (B) Contractile patterning occurs across spatial scales and levels of biological organization. Top: Myosin motor proteins aggregate within the actin cytoskeleton, for example, in the cytokinetic rings of fission yeast (adapted from Wollrab *et al*, 2016). Bottom: At much larger spatial scales, cells themselves can act as contractile elements within extracellular filament networks, leading to, for example, patterning of mesenchymal fibroblasts in collagen (adapted from Palmquist *et al*, 2022). Schematics represent the respective contractile units. (C) Linear stability analysis of a one‐dimensional contractile system on a ring with radius R reveals how the dominant modes at the onset of instability depend on the Péclet number, the ratio between convective and diffusive timescales (adapted from Palmquist *et al*, 2022). Dispersion relations are shown for three different Péclet numbers (left). The wavenumber with maximal growth rate increases as a function of Péclet number (right). (D) On a spherical domain with radius R, the ratio of the hydrodynamic length $L_{h}$ to the system size R determines the symmetry of the most unstable mode. This leads to distributions of contractile elements reminiscent of migrating cells for large $L_{h}$ (gray instability line) or dividing cells for smaller $L_{h}$ (blue instability line; adapted from Mietke *et al*, 2019a).

Contractile active stresses in combination with self‐induced convective flows give rise to a paradigmatic patterning mechanism, the contractile instability (Bois *et al*, 2011; Kumar *et al*, 2014; Hannezo *et al*, 2015; Palmquist *et al*, 2022). The intuitive idea underlying the instability is as follows: an active generator of mechanical stress, such as myosin, induces local contraction of the material, which produces a flow toward the region of contraction, bringing in more of the regulator and increasing the contraction further. This positive feedback loop can lead to regularly spaced regions of high contractility with associated flows toward them (Fig 4A).

Contractile instabilities appear in the cell cortex, where the cell itself is the domain on which the pattern forms and the contractile units are molecules. However, a cell can also be one of the small units that contribute to a larger, tissue‐scale pattern. Adherent cells typically exert active contractile stresses on their environment (Schwarz *et al*, 2002; Schwarz & Safran, 2013; Tanimoto & Sano, 2014). Similar to the way in which myosin molecules inside the actin network act as contractile elements, the cells themselves can constitute contractile units within a meshwork of extracellular matrix (ECM) filaments. As such, they can induce active rearrangements of the cell–ECM system, leading to contractile patterning at the multicellular level (Fig 4B, Harris *et al*, 1984; Shyer *et al*, 2017; Palmquist *et al*, 2022). Systems consisting of contractile cells embedded in extracellular matrix were, in fact, one of the earliest systems where mechanical forces were studied as a biological pattern‐forming mechanism (Oster *et al*, 1983; Harris *et al*, 1984).

To study contractile patterning theoretically, we write the continuity equation (equation 1) for the concentration of contractile particles c in one dimension:

(10) $$\partial_{t}c=D\partial_{\mathrm{xx}}c-\partial_{x}\left(\mathrm{vc}\right)+ℛ\left(c\right),$$

and include diffusive and convective fluxes and a reaction term. The velocity v is determined by the force balance equation (equation 3):

(11) $$\mathrm{\alpha v}=\partial_{x}\sigma ,$$

where the left‐hand side corresponds to an external frictional stress with friction coefficient α as before. For a contractile Newtonian fluid, the stress consists of two parts: $\sigma =\eta \partial_{x}v+\sigma_{A}$. Here, η is the viscosity, and the active stress $\sigma_{A}\left(c\right)$ depends on the concentration of the regulator. For contractile regulators, an increase in c leads to an increase in stress. Many studies assume a saturating relation between the active stress and the concentration of contractile particles (e.g., Bois *et al*, 2011; Mietke *et al*, 2019b). However, in the simplest case, valid for low concentrations, each unit (e.g., myosin molecule or contractile cell) imparts the same average stress ${\bar{\sigma }}_{A}$ to the material. Then, the active stress contribution depends linearly on the concentration of contractile particles, which we write relative to the average concentration $c_{0}$: $\sigma_{A}\left(c\right)={\bar{\sigma }}_{A}c/c_{0}$.

In the absence of chemical reactions or turnover—$ℛ\left(c\right)=0$—, the total amount of regulator is conserved (Bois *et al*, 2011; Palmquist *et al*, 2022). At steady state, the concentration is uniform at $c_{0}$, and there are no flows: $v_{0}=0$. Linear stability analysis of this steady state reveals that the growth rate ω of a spatial mode with wavenumber q obeys the following dispersion relation:

(12) $$\omega =-{\mathrm{Dq}}^{2}\left(1-\frac{\frac{{\bar{\sigma }}_{A}}{\mathrm{\alpha D}}}{1+\frac{\eta }{\alpha }q^{2}}\right).$$

The equation reveals that the existence of an instability only depends on two quantities. The ratio ${\bar{\sigma }}_{A}/\left(\mathrm{\alpha D}\right)$ is called the Péclet number and indicates the relative strength of the advective transport compared with the diffusive transport. The ratio $\sqrt{\eta /\alpha }$ is a length scale characterizing the spatial range of flows called the hydrodynamic length. There are solutions ω>0, corresponding to the emergence of patterns, only when the Péclet number is larger than 1 (Fig 4C). This shows that patterns form if the hydrodynamic flow induced by active stress is strong enough to overcome the stabilizing effect of random diffusive motion.

The fundamental pattern‐forming process is a self‐amplifying feedback loop where more regulator means more inward flow, bringing in even more regulators. This becomes clearer when writing the equation in the low‐viscosity limit, η→0:

$$\partial_{t}c=D\partial_{\mathrm{xx}}c-\frac{{\bar{\sigma }}_{A}}{c_{0}\alpha }\partial_{x}\left(c\partial_{x}c\right).$$

The local concentration evolves according to diffusion, the first term, which acts to smooth out concentration differences. The second term here describes that molecules move up their own gradient—the self‐amplifying feedback loop. If the latter outweighs diffusion, an instability appears. This mechanism, and the equations to describe it, are similar to pattern formation in chemotaxis and so‐called Keller–Segel models. In the case of chemotactic bacteria, the feedback loop is as follows: bacteria locally produce chemoattractant, bringing in more bacteria, producing more chemoattractant, and so forth. Again, this competes with the stabilizing effect of random motion—in this case of the organisms. Instabilities of this type occur across diverse biological contexts (see Painter, 2019, for a review).

In particular, the contractile fluid instability has been proposed to govern the arrangement of the feather follicles on the skin of birds (Shyer *et al*, 2017; Palmquist *et al*, 2022). The corresponding pattern‐forming system can be reconstituted *ex vivo* on a quasi‐one‐dimensional ring domain: when plated in drops on a collagen substrate, the dissociated dermal cells from chick embryos settle on the boundary of the droplet and undergo spontaneous patterning (Fig 4B). Over the course of aggregation, the cell–ECM layer in this system undergoes irreversible remodeling. The cells enmesh themselves, remodel, and induce irreversible rearrangements in the surrounding collagen, giving rise to an effective fluid‐like behavior of the cell–ECM layer on the timescale relevant for pattern formation (1–10 h). Pharmacological perturbations to the Péclet number and the hydrodynamic length yield changes in the number of aggregates and the timescale of patterning that are in good quantitative agreement with the predictions of the dispersion relation (equation 12). Increasing cellular contractility leads to faster patterning and more aggregates, and increasing hydrodynamic length leads to slower patterning into fewer aggregates (Palmquist *et al*, 2022). In this system, the final patterns are well described by linear stability analysis because the structures that appear immediately after the onset of the instability are stabilized by other mechanisms. *In vivo*, density differences are read out at early stages by beta‐catenin signaling (Shyer *et al*, 2017), while *ex vivo*, the depletion of material in between aggregates eventually appears to prevent sustained flows.

Another experimental system in which a contractile instability has been studied is the *Drosophila* tracheal tube. For this system, Hannezo *et al* (2015) included a reaction term of the form $ℛ\left(c\right)=\left(c_{0}-c\right)/\tau$ to describe turnover at a timescale τ. The linear stability analysis shows that here too, patterns emerge when advection dominates over other effects. Like diffusion, the turnover counteracts the self‐amplifying feedback loop of accumulation. Genetic and pharmacological perturbations to actin patterns in these cells also yield wavelength changes that match theoretical predictions.

Kumar *et al* (2014) extended the framework of contractile instabilities to two chemical species. One species upregulates the active stress and another downregulates it. In addition to stationary patterns, such a system also shows an oscillatory instability which leads to pulsating flow patterns. These pulsating patterns appear if the diffusion constants or the relaxation timescales for the upregulating and downregulating species are different.

Depending on the form of the reaction term, irregular spatiotemporal behavior is also possible. This has been described in the Keller–Segel model (Painter & Hillen, 2011): the inclusion of a quadratic growth term can lead to spatiotemporal patterns, with the irregular appearance and disappearance of high‐density regions. Hannezo *et al* (2015) also observed this in an extension of the model for actin dynamics in *Drosophila* tracheal cells, and linked it to the dynamic behavior of actin rings they found in one of the fly mutants.

Both the inclusion of multiple species and the addition of reaction terms can thus modify the patterns generated by contractile instabilities. On the other hand, patterns that are generated due to purely chemical interactions may be modified if the chemical species also induce flows. For example, if an activating chemical in a reaction–diffusion system also induces flows, the region in parameter space where patterns can be found become larger (Bois *et al*, 2011).

Contractile instabilities have been linked to the polarization of the cell cortex during cell migration, in particular where cells migrate without focal adhesions (Hawkins *et al*, 2011; Bergert *et al*, 2015; Liu *et al*, 2015; Ruprecht *et al*, 2015). For example, Hawkins *et al* (2011) study how contractile instabilities in the cortex of a spherical cell can lead to the spontaneous movement of the cell. Their model considers both actin and myosin concentrations in the cortex, recruitment of myosin from the cytoplasm, and the velocity field of the cortex. As in the study by Bois *et al* (2011), a linear stability analysis reveals that a contractile instability happens if the Péclet number is sufficiently large. An important difference is that the analysis is done on a spherical domain, rather than a one‐dimensional one. On a sphere, the pattern is characterized by unstable modes in the form of spherical harmonics (Figs 4D and 1C). In the model by Hawkins *et al* (2011), the most unstable mode at the onset of instability produces a polarized state where flows at the cell surface converge from one pole toward the opposite. The frictional coupling to the surroundings permits these active surface flows to generate a propulsion force that moves the cell forward. Indeed, migrating cells in many contexts exhibit gradients of myosin along their axis of motility, accompanied by rearward surface flows that drive propulsion in a friction‐dependent manner. The above formalism accurately and quantitatively predicts surface flows and cell velocities of migrating tumor (Hawkins *et al*, 2011; Bergert *et al*, 2015), immune (Liu *et al*, 2015), and embryonic progenitor cells (Ruprecht *et al*, 2015).

The dimension and shape of the domain on which the actin cortex is modeled are important for the resulting pattern, by constraining the spatial modes that can become unstable (see also Box 1, Fig 1C and section “Geometry‐dependent reaction‐diffusion patterns”). In real biological systems, contractile stresses can lead to deformations of this shape. Some studies have addressed how non‐uniform contractile stresses and flows change the system's shape (Ruprecht *et al*, 2015; Callan‐Jones *et al*, 2016), and how this in turn may feed back onto the flow pattern (Mietke *et al*, 2019a, 2019b). Ruprecht *et al* (2015) and Callan‐Jones *et al* (2016) study shape deformations due to cortical flows. As in the paper by Hawkins *et al* (2011), these studies were inspired by cell migration in three dimensions. The authors show that patterned active stress can produce an elongated cell shape, which corresponds to experimental observations.

The pattern of flow and contraction can thus lead to shape changes, but these, in turn, may influence the dynamics of surface concentrations. Mietke *et al* (2019b) studied the time evolution of coupled velocity and concentration fields on changing domains. The model equations describe the evolution of the concentration of a regulator of contractility and of the surface itself. The regulator is subject to advection and diffusion, which are influenced by the changing surface shape. The authors analyze the equations using linear stability analysis and numerical simulations and focus on (initially) spherical and tubular domains. On the spherical surface, instability can lead to polarization with high concentrations on one side, with a concomitant shape change. On the tubular domain, the instability can induce constriction of the tube with flow toward the narrow middle part. Moreover, oscillations and peristaltic waves are also possible. In a follow‐up work, Mietke *et al* (2019a) extend the formalism and couple the active surface to a bulk fluid in order to capture the interaction between the actomyosin cortex and the cytoplasm of a cell. Here, the contractile instability is accompanied by flows in the bulk, too. If the hydrodynamic length is small, the first mode to become unstable has a symmetric flow pattern with a ring of high concentration of regulator in the middle (Fig 4D), reminiscent of the contractile cytokinetic ring in dividing cells.

In summary, the physical principles of patterning through a contractile fluid instability apply across spatial and temporal scales spanning three orders of magnitude. Their theoretical analysis unveils connections between different levels of biological organization. Moreover, these works on contractile instability demonstrate how living systems can inspire new theoretical developments. The force‐generating, energy‐consuming properties of the actomyosin cortex, coupled with the cell's geometry, provide a system that does not have analogies in more traditional physical contexts.

## Buckling due to constrained growth

Contractile instabilities are characterized by self‐amplifying localized contractions. Another mechanism that can lead to mechanical instability—buckling—does not require localized contractility but can be generated by global growth or compression. Buckling is a familiar phenomenon from the everyday world, which arises if a thin object is subject to compressive forces, which lead to its bending. Buckling instabilities are also relevant for pattern formation in biological systems, where they can lead to regular wrinkles or folds. The formation of folds is important in many systems, including the gut (Savin *et al*, 2011; Shyer *et al*, 2013), the wrinkles of the brain (Llinares‐Benadero & Borrell, 2019), the development of the *Drosophila* wing (Tozluoğlu *et al*, 2019), and others (see Nelson, 2016, for a review). Buckling due to differential growth is a key mechanism in the determination of leaf shapes (reviewed in Guo *et al*, 2022). We do not discuss this in this review, but mechanical forces in general play an important role in plant morphogenesis (see Sampathkumar, 2020, for a review).

Other mechanisms besides buckling, such as localized contraction or non‐uniform growth, can also lead to folding (see Tozluoğlu & Mao, 2020, for an overview). In biological systems, it is not easy to distinguish which of these mechanisms is responsible for folding, even more so because they may act together. Recent advances have been made by Trushko *et al* (2020) in an *in vitro* system. There, the authors encapsulate a cell monolayer in an elastic alginate capsule. The growth of the monolayer leads to its folding. The folding did not occur in the absence of cell proliferation or of the encapsulating shell, pointing to buckling as the main folding mechanism. The setup also allows the measurement of material properties of the tissue, which the authors compared with continuum theory.

The buckling of beams and sheets is a classic problem in physics, but its application to biological systems has brought new elements to the theory. Rather than externally applied compressive stresses, in biological systems, it is often the growth of the tissue under confinement, or differential growth, that can lead to instability. The confinement can, for example, be due to a shell or surrounding tissues.

There is a wide range of literature on the mathematical description of growing systems (see Goriely, 2017; Ambrosi *et al*, 2019). Here, we will not go into the continuum mechanics of growing systems, but provide a simplified example based on energy considerations to show how buckling‐type instabilities can lead to pattern formation in constrained growing systems.

**Figure 5:**
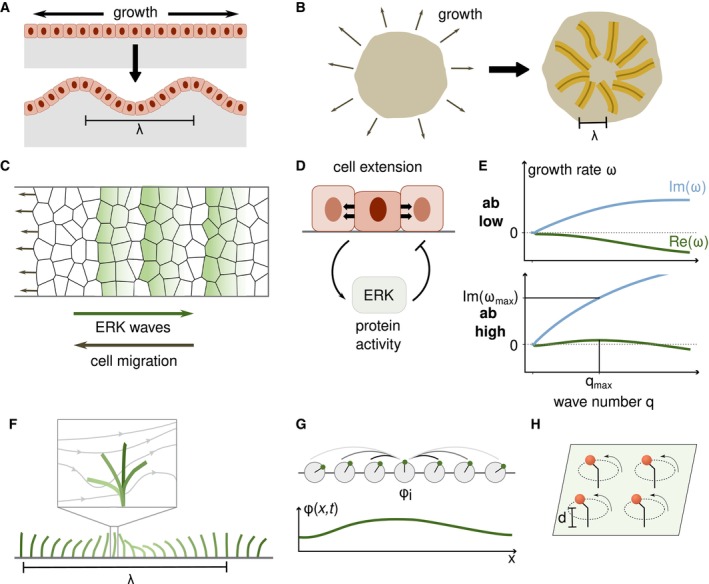
Buckling and waves (A) Cell growth in an epithelium attached to a substrate can lead to its buckling. (B) Bacterial biofilms that grow on a substrate can develop wrinkles due to a buckling instability (see also Fig 1A). (C) In epithelial sheets of MDCK cells, ERK waves propagate in the opposite direction of collective cell motion. (D) A negative feedback loop between cell length and ERK activity can lead to the emergence of mechanochemical waves in epithelial monolayers. (E) Dispersion relation for equation (15). For low feedback strength ab, the steady state is stable. Higher values of ab lead to an instability. The growth rate has an imaginary part, which corresponds to traveling wave patterns. The waves’ spatial and temporal frequency are determined by $q_{\max}$ and Imωmax_,_ respectively. Adapted from Boocock *et al* (2021). (F) Metachronal waves in ciliary arrays. Beating cilia generate flow fields. The fluid flows couple the motion of nearby cilia, which can give rise to coordinated motion and waves with a characteristic wavelength λ. (G) Synchronization of cilia and the emergence of metachronal waves can be modeled using coupled oscillator models. Each oscillator i is represented by its phase $\phi_{i}$, a number between 0 and 2π. The coupling strength can depend on the distance between the oscillators. If there are many oscillators, a continuum model can be appropriate, where the phase ϕ is a smooth function of position x (bottom). (H) The flow field generated by ciliary beating can be approximated by that of a sphere following an orbit above a plane. The distance of the sphere to the plane d determines the spatial decay of the generated flow field and therefore the form of the coupling function.

We illustrate the appearance of a buckling instability using a model for an epithelial monolayer attached to an elastic substrate with uniform cell growth (Fig 5A; inspired by Hannezo *et al* (2011) and Hannezo *et al* (2012), see also Brangwynne *et al* (2006) for a similar treatment of microtubule buckling). We assume a one‐dimensional system, where the height of the epithelium is given by h. In the weak bending approximation, also used in the section “Pattern formation by membrane curvature” for the cell membrane, the energy of the epithelium is given by

(13) $$F=\int_{0}^{L}\left(\frac{1}{2}\kappa {\left(\partial_{\mathrm{xx}}h\right)}^{2}+\gamma \left(1+\frac{1}{2}{\left(\partial_{x}h\right)}^{2}\right)+\frac{\beta }{2}{\left(h-h_{0}\right)}^{2}\right)\mathrm{dx}.$$

The first term is the bending energy, which is the same as for the discussion on biological membranes in the section “Pattern formation by membrane curvature”. The second term models the growth of the tissue, and can in this equation also be interpreted as a surface tension: the effect of cell proliferation is to locally increase the area—which can be modeled by considering a negative surface tension γ<0. The final term is the energy needed to deform the substrate. The parameter β is related to the substrate's stiffness and $h_{0}$ is a reference height. For simplicity, we consider only elastic penalties to changes in the height. As before, one can do an analysis of the stability of a flat epithelial layer, which shows that a pattern‐forming instability is possible when the growth of the layer overcomes the resistance to bending and the resistance to deformation of the substrate. The wavelength of the pattern depends on the mechanical properties of the tissues involved (Brau *et al*, 2013). The equation above is a simplified illustration; for an overview of different modeling approaches to this problem, see the paper by Almet *et al* (2020) and references therein.

Buckling instabilities may be followed by changes in signaling, as has been shown for example in the formation of villi in chick embryos (Shyer *et al*, 2015). There, the curved geometry induced by the folding leads to a patterned distribution of morphogens, which impacts the localization of stem cells in this system. There is thus an interplay among mechanics, geometry, and signaling.

Buckling due to constrained growth is not only seen in epithelial tissues but also in multicellular systems of another kind: bacterial biofilms. While a biofilm's size increases by the division and growth of bacteria, its substrate does not grow. This leads to the accumulation of stresses that result in a buckling instability and the appearance of wrinkles in the biofilm (Figs 5B and 1A). In a study by Yan *et al* (2019), these wrinkles develop into a striking star‐like pattern with a well‐defined wavelength. Classical theory for film–substrate systems predicts that the wrinkle wavelength should scale as ${\left(G_{f}/G_{s}\right)}^{1/3}$, where $G_{f}$ and $G_{s}$ are the shear moduli of biofilm and substrate. These moduli, which characterize a material's response to shear stresses, can be measured using a shear rheometer. The scaling relation is seen to hold true in a range of stiffnesses, but the two‐layer model is not an accurate description of the data for small $G_{f}$. Yan *et al* (2019) show that a model including a third layer is a better description of the data.

In bacterial biofilms, there can also be feedback between mechanics and biochemistry. Based on the pattern formation observed by Yan *et al* (2019), Fei *et al* (2020) set up a model of biofilm growth that also includes nutrient uptake and diffusion. Here, variations in nutrient concentration lead to inhomogeneous growth profiles, which influence the appearance of wrinkles. This system is another example of interactions among biochemistry, mechanics, and shape. The shape of the biofilm influences where nutrient uptake is highest, and nutrient availability determines the local growth rate, which then guides the shape of the biofilm.

Biofilms have recently been used to study other aspects of pattern formation in which mechanics plays a role. For example, mechanical instabilities are at the origin of verticalization and radial alignment, producing long range order in biofilms (Beroz *et al*, 2018; Nijjer *et al*, 2021).

The two examples of this section, epithelia and biofilms, illustrate how growth under confinement can lead to periodic patterning. Here, the patterning instability generates the tissue's or biofilm's shape. In turn, the shape can affect further mechanical or biochemical processes. These systems are another illustration of how mechanics and shape can introduce feedback loops that lead to the emergence of spatial patterns.

## Waves through mechanosensing

In the preceding sections, we mainly discussed mechanochemical systems in which feedback arises directly due to geometrical or mechanical effects on the distribution of constituents, such as curvature‐dependent localization (section “Pattern formation by membrane curvature”) or convective flows (section “Contractile fluid patterning”). At the multicellular level, however, the capacity of cells to sense mechanical properties and forces through special mechanosensory machineries and signaling pathways can become relevant for pattern formation. Mechanical forces sensed by the cell can lead, among others, to changes in gene expression, subcellular localization, or post‐translational modifications of proteins. In turn, these biochemical changes can affect the mechanical properties of a cell. In this way, mechanosensors can be part of mechanochemical feedback loops. Mechanosensing and mechanotransduction are important processes, but a more detailed discussion lies outside the scope of the current paper. We refer to the reviews by Chan *et al* (2017), Petridou *et al* (2017), and Kindberg *et al* (2020), and references therein, for more information.

In this section, we discuss one particular example where the ability of cells to respond to mechanical changes gives rise to pattern formation, involving an interplay between extracellular signal‐regulated kinase (ERK) activity and cellular shape. The pattern discussed in this section is varying not only in space but also in time, in the form of waves. Waves of ERK activity and contractility appear in systems of collectively moving cells (Aoki *et al*, 2017; Hino *et al*, 2020; Boocock *et al*, 2021). Collective cell motion is important for many biological processes in the development and maintenance of an organism, and a wide range of physical modeling approaches exists (see Alert & Trepat, 2020, for a review). Collectives of moving cells in epithelia show varied dynamics, including oscillatory motion and the propagation of mechanical waves (see, e.g., Dierkes *et al*, 2014; Zaritsky *et al*, 2014; Banerjee *et al*, 2015; Blanch‐Mercader & Casademunt, 2017; Peyret *et al*, 2019, and references therein). In sheets of epithelial migrating Madin–Darby canine kidney (MDCK) cells, waves of ERK activity propagate against the direction of the cell motion (Aoki *et al*, 2017; Figs 5C and 1A). These waves also appear in non‐migrating sheets of cells, where they are generated spontaneously in random directions (Boocock *et al*, 2021), and have been observed *in vivo* in mouse skin (Hiratsuka *et al*, 2015).

The protein kinase ERK is involved in different cellular pathways. Its role in collective motion has recently been addressed using FRET sensors detecting the activity of the protein (Aoki *et al*, 2017; Hino *et al*, 2020). Live imaging of moving cells and traction‐force microscopy have been combined with mathematical modeling to uncover how forces, motion, and signaling work together. In parallel with the increasing availability of spatiotemporal data, theoretical models of the system have been refined. The development of a simple continuum model by Boocock *et al* (2021), based on detailed experiments by Hino *et al* (2020), has elucidated how the interaction between signaling and cell contraction can lead to the appearance of spontaneous waves as well as organized collective motion.

The model describes an epithelial monolayer of cells as a one‐dimensional chain of overdamped springs. Using force balance, Boocock *et al* (2021) derive a continuum equation for the displacement of the cells:

(14) $$\tau_{r}\partial_{t}r=\partial_{\mathrm{xx}}r-\partial_{x}\ell^{0},$$

in which spatial scales have been normalized to the reference length of an individual cell. The time scale $\tau_{r}$ depends on the friction of the cells with the substrate and the strength of the spring‐like coupling.

The equation for r is complemented by one for the preferred cell length, $\ell^{0}$ and a third variable E that represents the local ERK activity. Hino *et al* (2020) found that ERK activity induces cell contraction. This can be modeled by stating that ERK levels tend to decrease $\ell^{0}$. Experiments have also shown that cell extension induces an increase in ERK levels, indicating that a mechanosensory process gives rise to feedback between the cell's mechanical and biochemical states (Fig 5D). However, how exactly the mechanical changes lead to a change in ERK activity is not fully understood (Hirashima *et al*, 2023). How this feedback is implemented thus becomes a modeling choice: Is ERK activation induced by absolute cell size, the cell strain, or strain rate? Boocock *et al* (2021), in an extended discussion of the model's assumptions, show that only the first choice leads to patterns in the model. This could provide a theoretical pointer toward the biological mechanism.

The equations for the model, with preferred cell length and ERK levels rewritten as a deviation from a basal level, are given by

(15) $$\begin{array}{ll} \tau_{r}\partial_{t}r & =\partial_{\mathrm{xx}}r-\partial_{x}\ell^{0},\\ \tau_{\ell }\;\partial_{t}\ell^{0} & =-\ell^{0}-\mathrm{aE},\\ \tau_{E}\partial_{t}E & =-E+b\partial_{x}r. \end{array}$$

The three constants $\tau_{r},\tau_{\ell }$ and $\tau_{E}$ are the timescales of evolution of the three variables. They are, like the Péclet number in the section “Contractile fluid patterning”, combinations of physical parameters such as friction constants and elastic coefficients. Importantly, these timescales correspond to directly measurable quantities: if one of the variables is perturbed from a steady state, the value of the corresponding τ indicates the timescale on which it relaxes back to that state. For example, an estimate for $\tau_{E}$ can be obtained by fitting an exponential function to measured ERK activity after mechanical stretching (Hino *et al*, 2020). The term −aE indicates that ERK activity induces contraction, and $b\partial_{x}r$ describes how cell extension induces ERK activation. This model does not contain detailed information about how ERK activity may biochemically induce cell contraction, nor how extension may lead to a rise in ERK levels. However, this information is not needed to make predictions about the large‐scale behavior of the system. All of the biochemistry is summarized in the parameters a and b. What is important is their sign, and their absolute value gives an indication of the strength of the feedback.

A linear stability analysis of the system reveals that the existence of an instability, and thus patterns, depends on the value of ab; the product of the feedback strengths (Fig 5E). In contrast to the instability that results in stationary patterns, the growth rate ω now has an imaginary component. The unstable mode is thus oscillatory in time and space, which indicates that the system supports traveling waves. The imaginary part of ω determines the temporal frequency of the waves. The oscillatory behavior is due to the negative feedback loop that is inherent in this system, even on a local level (Fig 5D): a cell's extension leads to ERK activation, which leads to a smaller preferred cell length, which leads to contraction, etc. Negative feedback loops are well‐known to generate oscillatory behavior, and combined with spatial coupling, they can lead to the appearance of traveling waves (Beta & Kruse, 2017).

Further analysis of the dispersion relation shows that the spatial wavelength and the temporal frequency of the waves close to the instability only depend on the three different timescales. Independent measurements of these timescales can thus be used to predict wavelength and frequency. The measurements for these timescales predict values of the wavelength and frequency in the range of the experimental observations. This correspondence between theory and experiment supports that the proposed wave‐generating mechanism is a plausible one.

The waves that are predicted by this theory have no preferred orientation: they can be induced by noise in the system and propagate in random directions. This recapitulates the observed random propagation of ERK waves in non‐migrating sheets of MDCK cells.

The directed propagation of ERK waves, which corresponds to cell migration in the direction opposite to the waves, can be explained by coupling the ERK–cell extension dynamics to cell polarity. Boocock *et al* (2021) investigate a model where cell polarity is induced by stress gradients. Cell polarity, in turn, affects the evolution of r. Simulations of this extended model show that the system can indeed produce collective cell migration. In this model, ERK waves that travel away from a free edge correspond to a persistent polarization of the follower cells, thereby guiding them to migrate collectively in the direction of the edge. Moreover, the model predicts that there is an optimal wavelength and frequency of ERK waves that produce the strongest average polarization. The ERK waves that have been observed in experiments have frequency and wavelength which roughly correspond to these optimal values, which suggests that the system is tuned for an optimal migration speed.

The ERK waves are an example where mechanics is one of the elements in a feedback loop that also includes biochemical components. Additionally, the patterns in this case vary in space as well as time, and a linear stability analysis provides information about the spatial and temporal scale of the patterns. Finally, the theory worked out by Boocock *et al* (2021) links the formation of patterns to a biological function, namely cell migration.

## Metachronal waves in arrays of hydrodynamically coupled oscillators

The mechanochemical waves described in the previous section arise from the instability of a uniform stationary steady state. However, waves can also be generated by different mechanisms. In systems where the individual units are already oscillatory, long‐range spatial patterns in the form of traveling waves can emerge due to coupling between different oscillators. If the individual units are moving appendages such as cilia or limbs, these waves are called *metachronal waves*. They are characterized by a phase shift between neighboring oscillators, which results in a typical wavelength λ (Fig 5F). In systems of cyclically moving appendages such as cilia or flagella, metachronal waves can generate locomotion of small organisms or fluid flows that transport particles (see, e.g., Byron *et al*, 2021, for an overview).

Metachronal waves are often studied using the theoretical framework of coupled oscillators. In coupled oscillator models, a complicated oscillating system is described by a single number: its phase (Fig 5G). As the oscillator traverses its cycle, the phase ϕ increases from 0 to 2π. Such models can describe a wide variety of systems—from the synchronization of flashing fireflies to mechanically connected metronomes (Pikovsky *et al*, 2003).

A general model of interacting oscillators reads

(16) $$\frac{d}{\mathrm{dt}}\phi_{i}=\omega_{i}+\sum_{j}G\left(|r_{i}-r_{j}|\right)H\left(\phi_{i},\phi_{j}\right),$$

in which $\phi_{i}$ is the phase of oscillator i, located at position $r_{i}$ (Fig 5G). Here, $\omega_{i}$ is the frequency of the unperturbed oscillator and the function H describes how the coupling between oscillators depends on their phases. The function G describes how the strength of the coupling depends on the distance between the oscillators.

Equation (16) is an equation for discrete oscillators, which is the form mostly used for studying metachronal waves. Some studies, however, use continuous phase equations, where the phase is given by a field $\phi \left(x,t\right)$ (Fig 5G, Chakrabarti *et al* (2022) and Quillen (2023)). Interestingly, continuous phase equations with non‐local coupling also appear in models of collections of neurons, where long‐range interactions are due to the spatial extent of axons (e.g., Crook *et al*, 1997). A continuous version of equation (16) reads

(17) $$\partial_{t}\phi \left(x,t\right)=\omega \left(x\right)+\int dyG\left(\left|x-y\right|\right)H\left(\phi \left(x,t\right),\phi \left(y,t\right)\right).$$

A uniformly oscillating system is a solution to this equation with $\phi \left(x,t\right)=\mathrm{\omega t}$. It is, similar to the examples in the other sections, possible to do a linear stability analysis of this state. Moreover, depending on the functions G and H, it is possible to examine analytically the existence and stability of traveling wave states of the form $\phi \left(x,t\right)=\mathrm{qx}+\mathrm{\omega t}$, where q is the wavenumber. Equations like equation (17) can admit multiple distinct wave solutions, and which waves are more likely to be seen in systems described by these mathematical models is an ongoing topic of study (see, e.g., Wiley *et al*, 2006; Solovev & Friedrich, 2022). Moreover, theoretical studies of non‐locally coupled oscillators showed that these models support other types of behavior, such as the coexistence between synchronized regions with randomly oscillating oscillators (e.g., Kuramoto & Battogtokh, 2002; Abrams & Strogatz, 2004). This raises the intriguing question of whether real biological systems, such as ciliary arrays, could also show these kinds of behavior (see Gilpin *et al*, 2020, for a more extended discussion).

A prominent biological example of metachronal waves is found in arrays of cilia. Each cilium is a hair‐like appendage that exhibits periodic motion due to the activity of internal molecular motors (recent overviews of the physics of cilia and flagella are given by Wan, 2018; Gilpin *et al*, 2020). This motion generates a fluid flow that influences neighboring cilia (Fig 5F). Due to the relatively long‐ranged nature of fluid flows, the interactions between cilia are non‐local. This illustrates how mechanical features can lead to types of coupling that are different from the coupling produced by, for example, diffusion, or local mechanical coupling such as in epithelial tissues.

Understanding the physics of metachronal waves on ciliary arrays is a multiscale problem from the molecular to the organismal scale (Chakrabarti *et al*, 2022). General scaling arguments from hydrodynamics, however, can provide insights into the spatial interactions between cilia because the flow field at a certain distance from the cilium can be well approximated by the flow field of a sphere moving on a cyclic orbit (Brumley *et al*, 2014; Fig 5H). Flow fields decay with distance r as 1/r for free spheres and as $1/r^{3}$ for spheres close to a wall (e.g., Happel & Brenner, 1983; Vilfan & Jülicher, 2006; Niedermayer *et al*, 2008), implying a similar scaling for the coupling between oscillators. These hydrodynamic arguments provide the basis for some of the coupled oscillator models (Fig 5G), in which the hydrodynamic effects between the cilia determine the functions G and H in equation (16). For example, Uchida & Golestanian (2010) apply multiple approximations to a hydrodynamic model and derive a reduced model with $G\left(r\right)=A/r^{3}$ and $H\left(\phi_{i},\phi_{j}\right)=\sin\left(\phi_{j}-\phi_{i}+\delta \right)$, in which the coupling strength A depends on properties of the fluid surrounding the cilia. Importantly, the range of spatial coupling determines the stability of metachronal waves (Wollin & Stark, 2011; Brumley *et al*, 2016).

The mechanical aspect relevant to metachronal waves is mainly the hydrodynamic coupling. However, the shape of the domain on which the ciliary arrays are present can also be important. In the natural world, ciliary arrays often appear on non‐flat domains such as spheres. The question of how different domain shapes affect the synchronization of coupled cilia has recently been addressed theoretically (e.g., Westwood & Keaveny, 2021, and references therein).

Ciliary arrays are not the only biological systems that show metachronal waves. They also appear on the level of multiple organisms, as has been described by Peshkov *et al* (2022). In this study, the authors observe swimming nematodes (*T. aceti* or vinegar eels, see also Fig 1A). These small worms, when confined in liquid droplets, synchronize their undulating motion when their density is high. The authors also describe a model based on coupled oscillators (Quillen *et al*, 2021b), where they assume that the worms inhibit the motion of their neighbors when they overlap—effectively a mechanical coupling. This type of synchronization of oscillatory motion has also been recovered with robots (Zhou *et al*, 2021, Box 2). These examples show how even the most basic forms of “mechanics”—pushing through contact—can lead to patterns.

Finally, the understanding of metachronal waves through theoretical modeling has also helped in the design and construction of artificial carpets of cilia, whose metachronal waves can be harnessed to generate desired fluid flows and transport (see, e.g., Ul Islam *et al*, 2022, for a review).

## Conclusions

The importance of mechanics and shape in biology has been appreciated for a long time (Thompson, 1917). However, more recently, experimental advances have allowed quantitative measurements of forces, flows, and shapes of cells and tissues. It is now clear that mechanical and geometrical properties of cells and tissues play an essential role in biological organization and function across diverse contexts and scales (e.g., Gross *et al*, 2017; Naganathan & Oates, 2017; Hannezo & Heisenberg, 2019; Maroudas‐Sacks & Keren, 2021; Bailles *et al*, 2022; Valet *et al*, 2022; Dullweber & Erzberger, 2023). Feedback between mechanical changes, shape dynamics, and biochemical or genetic regulatory processes leads to the formation of mechanochemical patterns.

In this review, we highlighted theoretical developments, mostly from recent years, on mechanical and mechanochemical pattern formation. We discussed in particular how systems on spatial scales spanning 1‐10000μm and levels of biological organization from subcellular to organismal are governed by the same physics. The fact that these systems can be described by the same equations illustrates the generality of some of the outlined mechanochemical pattern‐forming mechanisms. Theoretical approaches have been crucial to uncover these common mechanisms. Conversely, mechanochemical pattern formation has spurred the development of new theories, leading to new insights into the physics of complex systems (Prost *et al*, 2015; Bowick *et al*, 2022; National Academies of Sciences, Engineering, and Medicine, 2022; Hallatschek *et al*, 2023).

The main theoretical tool we focused on is linear stability analysis. Even though the premise of this approach is that the system is close to a steady state, it proves to be remarkably useful in understanding pattern formation in general settings. Yet, a more complete understanding of patterns, including their long‐time behavior, can require additional theoretical tools or computer simulations (Box 2). In many living systems, processes are coupled across different spatiotemporal scales and levels of biological organization. For example, in some cases, pattern formation in a tissue can be described by continuum equations, treating the cells as microscopic units whose dynamics can be described by fields, but sometimes the cell's individual features matter too, in which case a model with cells as discrete units may be more appropriate. In particular, when microscopic units adapt and respond to changes at larger scales, behaviors can arise that are not seen in passive physical systems, motivating theoretical approaches that not only explain how large‐scale phenomena arise from microscopic dynamics but which can also capture the effects of scale‐crossing bidirectional interactions.

In this review, we have discussed only deterministic models and did not touch upon the role of noise and stochasticity. However, noise is always present in biological systems, and how noise is involved in giving rise to structures or processes with biological functions is an intriguing question, also in the context of pattern formation. For example, stochastic effects can lead to large‐amplitude patterns in systems that, when considered deterministically, exhibit a stable homogeneous steady state (e.g., Biancalani *et al*, 2017; Karig *et al*, 2018).

Forces, flows, and geometry can induce feedback on time and length scales very different from those accessible to molecular mechanisms. Coupling biological regulatory mechanisms to mechanics or geometry can therefore enable dynamics or patterns difficult to attain otherwise. Generally, rich dynamical phenomena are the result of feedback terms in the governing equations. In biochemical systems, such feedbacks typically involve multiple interactions and non‐linearities. Importantly, however, mechanochemical coupling can lead to complex phenomena with no cost or even with an improvement to the parsimony of our theoretical understanding. Cells do not escape the laws of physics, or in other words, mechanics and shape are always there, and including them in theoretical descriptions of biological systems often leads to simpler explanations for observed phenomena. In fact, neglecting fundamental physical properties can require complicated implicit assumptions that are unjustified, unrealistic, or even impossible. Non‐linearities are required for pattern formation, and in models that focus exclusively on biochemical effects, these must be postulated to arise purely from molecular interactions. In the Schnakenberg equations, for example (equations 19, Box 1), the non‐linearity arises from a trimolecular reaction term with a single rate constant. For mass action kinetics, this is unrealistic since it requires the simultaneous collision of three molecules with a non‐negligible probability. Many biochemical pattern‐forming models make assumptions of—often unknown—reactions that are summarized by a few non‐linear terms. Taking the—typically known—effects of mechanics and geometry into account can make invoking additional biochemical complexity unnecessary.

The inevitability of geometry and mechanical interactions raises the question of how patterns that rely on mechanics have evolved, and whether mechanical pattern formation is more robust compared to genetic or biochemical patterns (Box 2). It will be interesting to investigate—for specific cases and across different contexts—whether patterns or their functions become less vulnerable to variations in parameter values when they are generated by mechanochemical feedback mechanisms. We expect that a deeper understanding of these questions, and progress toward answers, will require a strong interaction between experimental advances and theoretical approaches.

## Author contributions

**Jan Rombouts:** Conceptualization; formal analysis; funding acquisition; visualization; writing – original draft; writing – review and editing. **Jenna Elliott:** Conceptualization; formal analysis; visualization; writing – original draft; writing – review and editing. **Anna Erzberger:** Conceptualization; supervision; funding acquisition; visualization; writing – original draft; writing – review and editing.

## Disclosure and competing interests statement

The authors declare that they have no conflict of interest.
